# *BioNet*: A Python interface to NEURON for modeling large-scale networks

**DOI:** 10.1371/journal.pone.0201630

**Published:** 2018-08-02

**Authors:** Sergey L. Gratiy, Yazan N. Billeh, Kael Dai, Catalin Mitelut, David Feng, Nathan W. Gouwens, Nicholas Cain, Christof Koch, Costas A. Anastassiou, Anton Arkhipov

**Affiliations:** 1 Allen Institute, Seattle, WA, United States of America; 2 University of British Columbia, Vancouver, BC, Canada; Universitat Pompeu Fabra, SPAIN

## Abstract

There is a significant interest in the neuroscience community in the development of large-scale network models that would integrate diverse sets of experimental data to help elucidate mechanisms underlying neuronal activity and computations. Although powerful numerical simulators (e.g., NEURON, NEST) exist, data-driven large-scale modeling remains challenging due to difficulties involved in setting up and running network simulations. We developed a high-level application programming interface (API) in Python that facilitates building large-scale biophysically detailed networks and simulating them with NEURON on parallel computer architecture. This tool, termed “BioNet”, is designed to support a modular workflow whereby the description of a constructed model is saved as files that could be subsequently loaded for further refinement and/or simulation. The API supports both NEURON’s built-in as well as user-defined models of cells and synapses. It is capable of simulating a variety of observables directly supported by NEURON (e.g., spikes, membrane voltage, intracellular [Ca++]), as well as plugging in modules for computing additional observables (e.g. extracellular potential). The high-level API platform obviates the time-consuming development of custom code for implementing individual models, and enables easy model sharing via standardized files. This tool will help refocus neuroscientists on addressing outstanding scientific questions rather than developing narrow-purpose modeling code.

## Introduction

Neuroscience is undergoing a profound revolution in the sensitivity and throughput of its experimental methods, which are beginning to yield large, systematic datasets relating to the structure and activity of brain circuits [1–4]. This revolution has spurred a growing interest in the neuroscience community towards the development of large-scale biophysically detailed network models as a means for integrating diverse sets of experimental data and discovering the principles that underlie functions of neuronal circuits. Although several such large-scale models have been developed [5–9], data-driven modeling studies at large scale remain very challenging, largely because adequate software tools are not yet available.

The development of simulation environments such as NEURON [10,11], GENESIS [12] and more recently Arbor [13] has greatly facilitated the process of setting up and running biophysically detailed network simulations. These environments allow users to develop models using neurophysiological concepts (e.g., dendritic sections, synapses, ionic conductances), while seamlessly handling numerical tasks such as solving the cable equation with ionic membrane mechanisms and tracking spike event arrivals to cells in the network. In particular, NEURON has become a widely used tool, due to its capability to simulate the activity of biophysically detailed cells and their networks, support of simulations on parallel hardware [14,15], and the implementation of a Python interface [16].

Despite these advances, modeling large-scale networks of biophysically detailed cells with NEURON still requires a substantial coding effort and detailed knowledge of the simulation environment. This is due to NEURON providing users with a scripting rather than high-level functionality interface for setting up and running network simulations. It is thus left to individual modelers to write code in the NEURON environment to set up and run their simulations, which can be a very laborious task. Typically, computational neuroscientists implement this functionality tailored to a particular model and for specific questions. Adopting such tailored simulation codes to different models usually requires extensive modification, hindering sharing and reuse of models and code for running simulations.

The challenges of model sharing and reuse are well recognized in the computational neuroscience community and a number of software tools facilitating network modeling with NEURON have been introduced (e.g., NeuroConstruct [17], PyNN [18] and NetPyNE (neurosimlab.org/netpyne/)). Motivated by the specific applications and operational philosophies, each suite is best suited for addressing different kinds of modeling problems. In turn, modeling large-scale networks (~100,000 neurons) of biophysically detailed cells (with hundreds of compartments each) brings its own specific considerations arising from computational demand for simulating complex models.

Development of large-scale biophysically detailed models typically requires a multi-step incremental procedure (e.g., creating populations of cells, adding an external input, adding recurrent connectivity) in conjunction with intermediate simulation and analysis stages. Each of these stages may be computationally expensive (e.g., for a network of ~100,000 neurons with sophisticated connectivity rules building the adjacency matrix can take several days when executed on a single processor), and therefore for large networks it becomes imperative to be able to save intermediate results of each stage. This calls for a modular incremental workflow where different modeling stages–building, simulation, and analysis–can be performed independently. Thereby, a constructed model could be saved to files, if necessary modified and elaborated, and later used as input to the network simulator and analysis tools. Furthermore, biological networks exhibit highly heterogeneous properties of cells and connections, requiring the software to be flexible enough to support custom user functionality for defining the composition of the network. At the same time, it should allow inexperienced users to perform standard operations without having to become experts in a particular scripting environment. Finally, large-scale modeling requires a versatile, modular, query-friendly and computationally efficient network description format for storage of model parameters enabling an interface between different modeling stages.

With these aims in mind, we developed BioNet, a modular Python application programming interface (API) facilitating building and simulation of large-scale biophysically detailed networks. The builder component of the API provides functionality for creating networks and saving the detailed network description to files based on user specified properties of cell types and connectivity rules. The simulator component implements an interface to NEURON and provides the functionality for loading the network description from files, instantiating a model on parallel computer architecture, setting up the simulation parameters, computing the desired observables (e.g., membrane voltage, extracellular potential) using NEURON and saving the simulation results. Thus, BioNet on the one hand lowers the barrier for conducting modeling studies of biophysically detailed networks to scientists with very basic programming skills, while also accelerates model development for seasoned computational neuroscientists. BioNet is publicly released as a component of a modeling tool suite called Brain Modeling Toolkit (version 0.0.5) with the code hosted at github.com/AllenInstitute/bmtk and documentation and tutorials hosted at alleninstitute.github.io/bmtk/.

## Materials and methods

### Modeling paradigm

Modeling large-scale biophysically detailed networks involves several consecutive stages. Modeling usually starts with a conceptual (high-level) definition of the network under study: which brain regions to include, what level of detail to implement for cells and synapses, and how to treat their interactions. Often one can distinguish the explicitly simulated network model, which may include recurrent connections, from the feed-forward external inputs that are not treated explicitly in the simulation. The external inputs are influencing the neurons in the network, but the network does not influence the external inputs. All information about the external input can be computed separately from the network simulation, such that, for instance, their spike trains are pre-computed and saved, and are then loaded from files to drive the network simulation.

**Fig 1:**
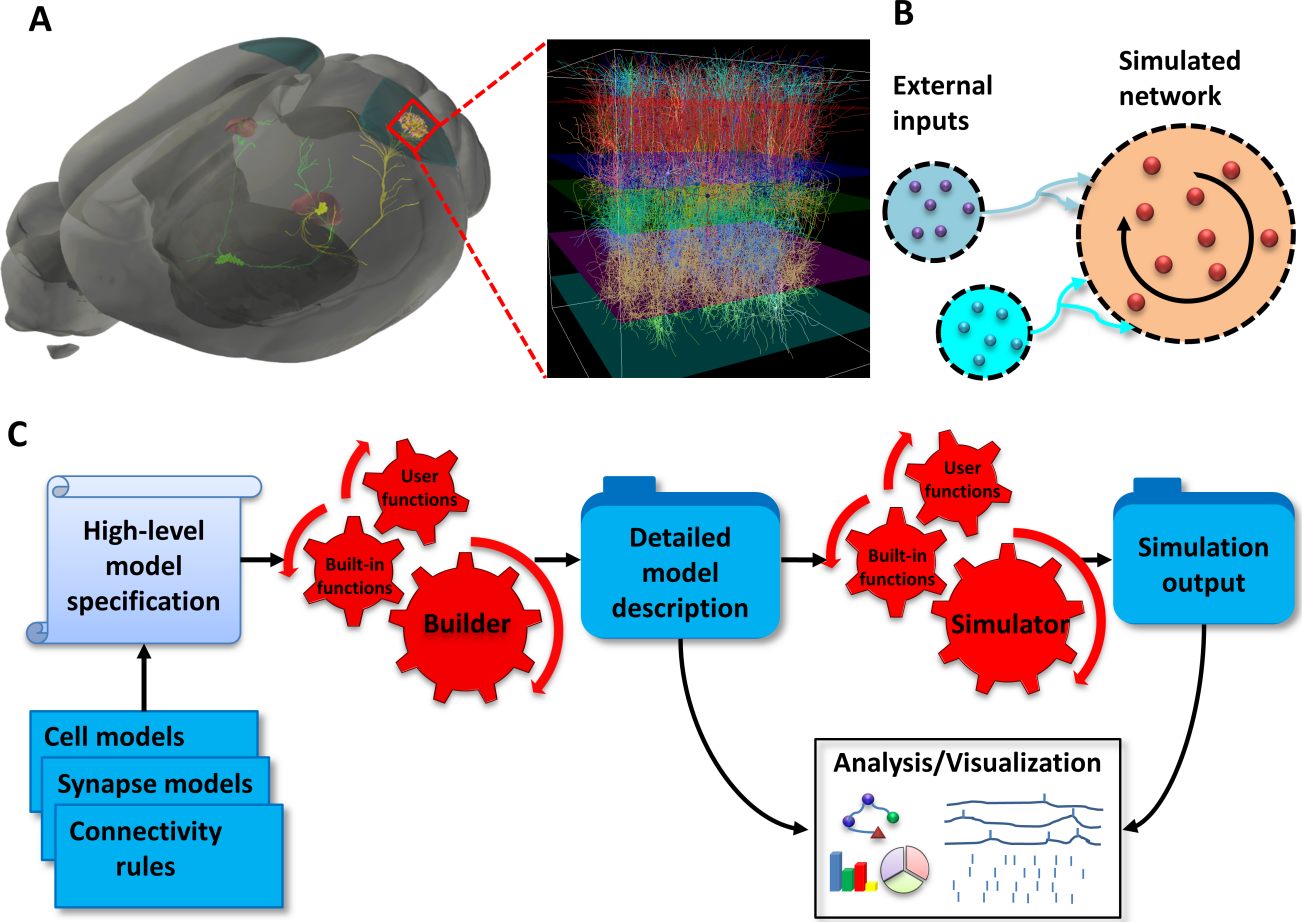
Modeling paradigm. (**A**) Modeling a large-scale network of neurons of the mouse primary visual cortex (area V1) as an example. Anatomically, retinal ganglion cells project (green), among other targets, to the Lateral Geniculate Nucleus (LGN) of the thalamus (red). The LGN cells project to V1 (zoomed-in view). In this example, the LGN activity is pre-generated and treated as external input, whereas V1 is simulated explicitly (“simulated network”). (**B**) Conceptual representation of the network model as a set of populations of external sources providing feedforward inputs (blue circles) and the simulated network (orange circle), which is recurrently interconnected. (**C**) Stages of the modeling workflow: The model components (blue stack) and the high-level model specification (scroll) are passed to the builder (red gears) to produce detailed model description as a set of files (blue folder). This network description serves as an input to the simulator (red gears) to produce the simulation output as a set of files (blue folder). In turn, the outputs from the simulator and builder serve as input for the analysis and visualization tools (white board).

For instance, when modeling activity in the mammalian visual system (Fig 1A) retinal ganglion cells can be viewed as providing feed-forward external input to the Lateral Geniculate Nucleus (LGN) of the thalamus, which in turn links to the primary visual cortex (V1). In this case both LGN and V1 cells must be included as a part of a simulated network in order to study their interactions. Alternatively, if the V1 to LGN feedback does not need to be modeled directly, we may treat the LGN as a feed-forward relay to the V1 network (Fig 1B). The LGN activity can then be pre-computed (or taken from experimental recordings, see, e.g., [19]) and treated as external input to the simulated V1 network model.

Subsequently, we proceed with defining the components (e.g. cell types/models, number of cells, connectivity rules and synaptic types/models) of the external inputs and the simulated network (Fig 1C). These definitions are used to create individual instances of cells, connections and their specific parameters constituting a detailed description of a network model. Once all the descriptions of a network are supplied, the model may be simulated to produce network activity. The network activity then may be analyzed in combination with the detailed description of the network.

In the case of large-scale biophysically detailed networks, the building stage typically involves a multi-step procedure which can be computationally expensive. It is thus highly desirable to explicitly separate the building and simulation stages of the modeling. Thus, the building stage produces an explicit description of the network and saves it to disk for subsequent simulations and analysis. This enables multiple simulations to be run for the same network build–but with different run-time parameters such as external input, duration of simulation or what data to be saved (e.g. membrane voltage, intracellular calcium or extracellular potentials on different electrode layouts).

### Model components

**Fig 2:**
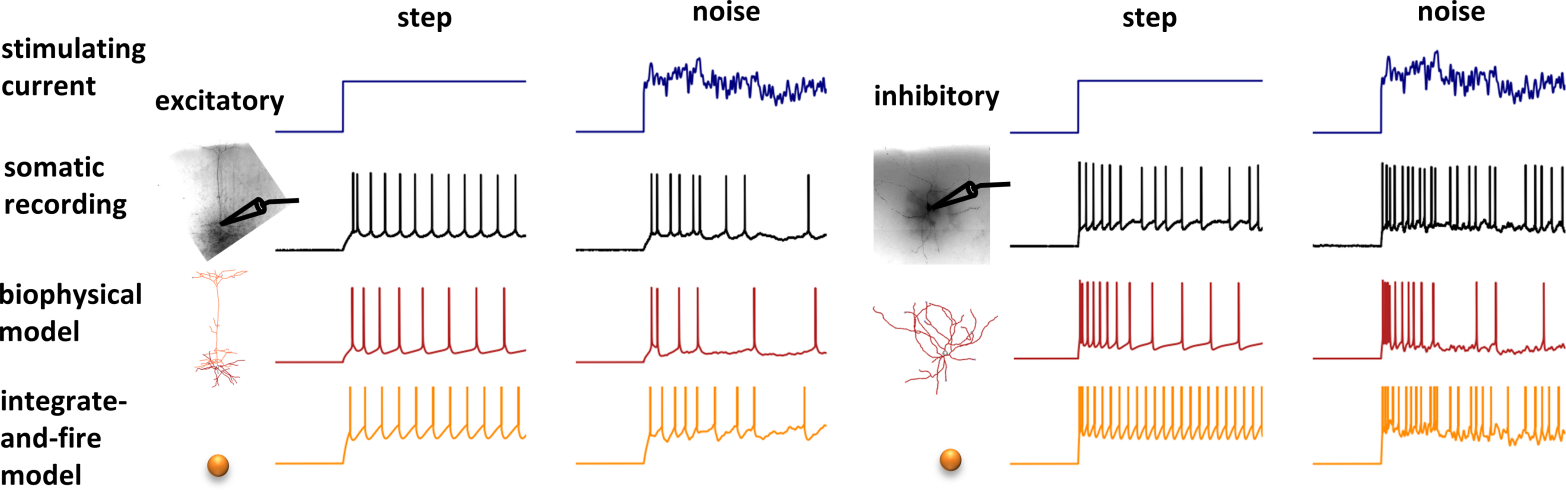
Examples of supported levels of detail for cell models. Experimental somatic recordings (black) in response to stimulating current (blue) can be modeled with BioNet at multiple levels of resolution (as long as they are supported by or can be implemented in NEURON). Here, two extremes are shown: biophysically detailed (red) and point leaky integrate-and-fire (LIF) models (orange). Example recordings and fits are from the Allen Cell Types Database.

To build a BioNet model, users must decide on the component building blocks: models of individual cells and synapses corresponding to different cell and synapse types, as well as the connectivity rules between different cell types. BioNet facilitates simulations of neuronal activity at many levels of biophysical detail, ranging from realistic morphologies with time- and voltage-dependent ionic conductances to highly simplified integrate-and-fire point models (Fig 2). To utilize a particular model type of a cell/synapse, the user simply needs to specify a Python function returning an instance of a NEURON’s cell or synapse. BioNet comes with several built-in functions that users may readily adopt to their models. Other custom models may be included by creating functions that may either fully implement biophysical and morphological properties, load them from files (e.g., JSON, SWC), or include a call to a NEURON’s legacy HOC model. This capability gives users the flexibility of employing both built-in NEURON models of cells (e.g., IntFire) and synapses (e.g., Exp2Syn), as well as a variety of published models, such as those from ModelDB [20] and the Allen Cell Types Database [21–23].

Similarly, in order to specify the properties of connections between cells, the user needs to specify a function that determines connections between the cells based on user defined connectivity rules (e.g., distance dependence, orientation preference dependence, etc.). Generally, any combination of the cell’s properties could be used to define the connectivity rules between two cells. For convenience, our builder toolkit provides a number of connectivity functions that are typically utilized in network modeling and serve as examples for creating custom connectivity rules.

## Results

Below we describe the operational workflow and functionality of our toolkits for building and simulation of large-scale biophysically detailed networks. We focus primarily on a user experience that does not require changing the source code, although, as the package is open-source, users can freely modify it or add functionality by consulting the online documentation.

### Building networks

The builder toolkit facilitates constructing network models based on high-level user specification of a network composition from the available modeling components. In order to efficiently support the development of highly heterogeneous multi-purpose models at large scale, the builder was designed to satisfy several conditions. First, it provides functionality for assigning properties of cells and their connections according to user-defined rules. Second, it enables a step-by-step workflow where a portion of a model could be developed and saved to files and then subsequently loaded for further refinement. For instance, multiple feed-forward inputs and/or recurrent connections may be constructed in stages or even independently. This capability enables constructing different components of one model by a team of scientists and then merging them into a single interconnected network model. Third, an essential design consideration of a builder is that small changes to the model composition or parameters must require small effort on a user’s part. For instance, changing biophysical properties of a cell type in an existing network will not affect connectivity properties of the network. Thus, when modifying such properties, the builder will not need to load or re-generate connectivity. This capability facilitates model refinement and adoption by different users.

**Fig 3:**
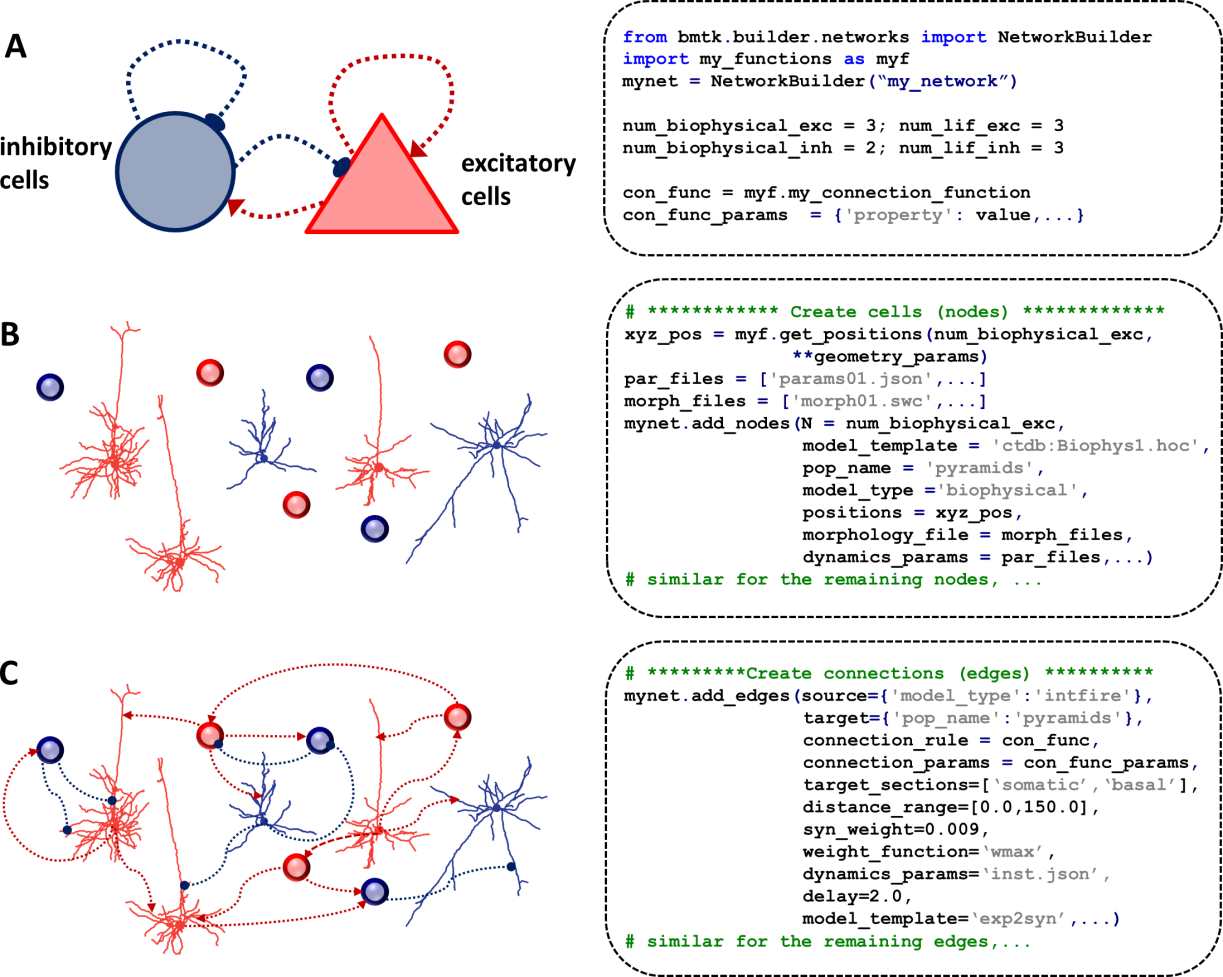
Building networks. (**A**) High-level specification of a simple example network (left) and corresponding builder API commands (right). The model is composed of two cell types: inhibitory (blue) and excitatory (red), which exchange connections both across and between the cell types. The API commands define the number of cells of each type to be created, connectivity rule (con_func) to use and associated parameters (con_func_params) as well as additional edge parameters (edge_type_params). (**B**) Illustration of creating cells (left) where each cell type may include both biophysical (morphological reconstruction) and LIF models (circles). The corresponding API commands for adding nodes for the biophysically detailed subset of excitatory populations are illustrated on the right. Here we specify the number of nodes to be created (N), a type of a model (model_type), the dynamical cell models (model_template) and the corresponding model parameters (dynamics_params), morphologies (morphology_file), and positions of cell somata (positions) that were computed with a user-defined function. (**C**) Illustration of connecting the cells into a network (left) and the corresponding API commands for adding a particular subset of connections (right). Here, the cells satisfying the query for both the source and target nodes will be connected using a function (connection_rule) with parameters (connection_params). The additional edge_type attributes are shared across the added edges and include the synaptic strength (syn_weight), function modulating synaptic strength (weight_function), dynamical synaptic model (model_template) and corresponding parameters (dynamics_params), a conduction delay (delay), as well as the locations where synapses could be placed on a cell (target_sections, distance_range).

Building a network starts with specifying the high-level composition of a network: the cell types and the number of neurons of each type as well as the user functions defining properties of the cells and the connectivity rules (Fig 3A). The builder then interprets these rules to create a specific instantiation of a network that can be saved to files and subsequently loaded by BioNet to be simulated. Other instantiations can be generated by re-running the building stage with different random seeds. Recognizing that a model network is a graph, we can construct networks by first adding the graph nodes (cells) and then adding edges (connections) between the nodes.

The nodes are added to the network by invoking the add_nodes(…) command (Fig 3B). One can use it to add individual nodes, or groups of nodes that share common properties. The key-value pairs in the argument of a function are the node property names and the corresponding assigned property values. Each invocation of add_nodes(…) command creates a corresponding node type defined by the properties shared across the added nodes. Users can assign such common properties by assigning a single value to the property name (e.g., pop_name = “pyramid”). Alternatively, if a property has different values for each instance of the added node, user must specify an array of values with the size equal to the number of added nodes (e.g., positions = xyz_pos). The number of properties and their names are not restricted by the builder and the user is free to specify arbitrary key-value properties. However, to use the BioNet simulator, some parameters are mandatory. These include: (1) model_type (e.g., “biophysical”, “intfire”); (2) model_template defining the dynamics of a model and the corresponding parameters dynamics_params; (3) params_file/morphology_file specifying the biophysical parameters of a model. Additional properties of the nodes can be added to be used when defining connectivity rules (e.g., positions) or for bookkeeping purposes (e.g., population_name).

Subsequently, the nodes are connected with the add_edges(…)command (Fig 3C). The cells involved in a connection are selected by specifying queries for the properties of the source and target nodes. Similar to nodes, each invocation of add_edges(…)command creates an edge type that can share some property defining the edge type (e.g., dynamics_params = 'GABA_InhToExc.json'). Alternatively, if the connection properties are specific to the individual connected pair, then the builder will find connected pairs using the connection_rule function with parameters in the connection_params dictionary and assign the desired properties to the individual connections. Users can specify any number of additional connection parameters using key-value pairs; however, to use with the simulator, some parameters are mandatory just as in the case of the nodes. These include: (1) model_template defining the dynamics of a model and the corresponding parameters dynamics_params; (2) syn_weight specifying the synaptic strength together with the function weight_function modulating synaptic strength; and (3) a delay specifying a conduction delay. In addition, depending on the way the connectivity is defined, the user may need to specify target_sections (”apical”,”basal”,”somatic” and”axonal”) as well as distance_range (minimal and maximal distances from the soma on target_sections) for establishing the synapse placement.

Although previous community efforts resulted in the development of network description approaches (e.g., NeuroML [24] and NineML [25]), little effort has focused thus far on efficient description of large-scale networks, where data size, memory efficiency for simulations on parallel hardware and the ability to readily query and modify network parameters are the dominant determinants in the choice of the data structures. As a result, in the existing approaches the files describing network parameters (e.g., connectivity) tend to use unnecessarily large amounts of disk space and computer memory for representing realistic brain networks. Furthermore, modifying or adding network properties may be a computationally expensive task (for large networks), potentially requiring loading and rewriting the entire network description. BioNet offers a solution to these issues by storing parameters of the nodes and edges in separate but related binary tables (thus, saving disk space), enabling highly independent representation of network parameters. In addition, the parameters common to a group of nodes/edges can be grouped to define node/edge types and stored in separate node_type/edge_type tables enabling a parsimonious network description. Such a “type-instance” representation enables easy modification of the properties of the node/edge types without necessarily needing to iterate through individual nodes/edges, saving much computational cost for large networks. For instance, one can easily change properties of synapse types by editing a small file containing edge type properties, without having to modify or regenerate the huge adjacency matrix file.

### Running simulations

**Fig 4:**
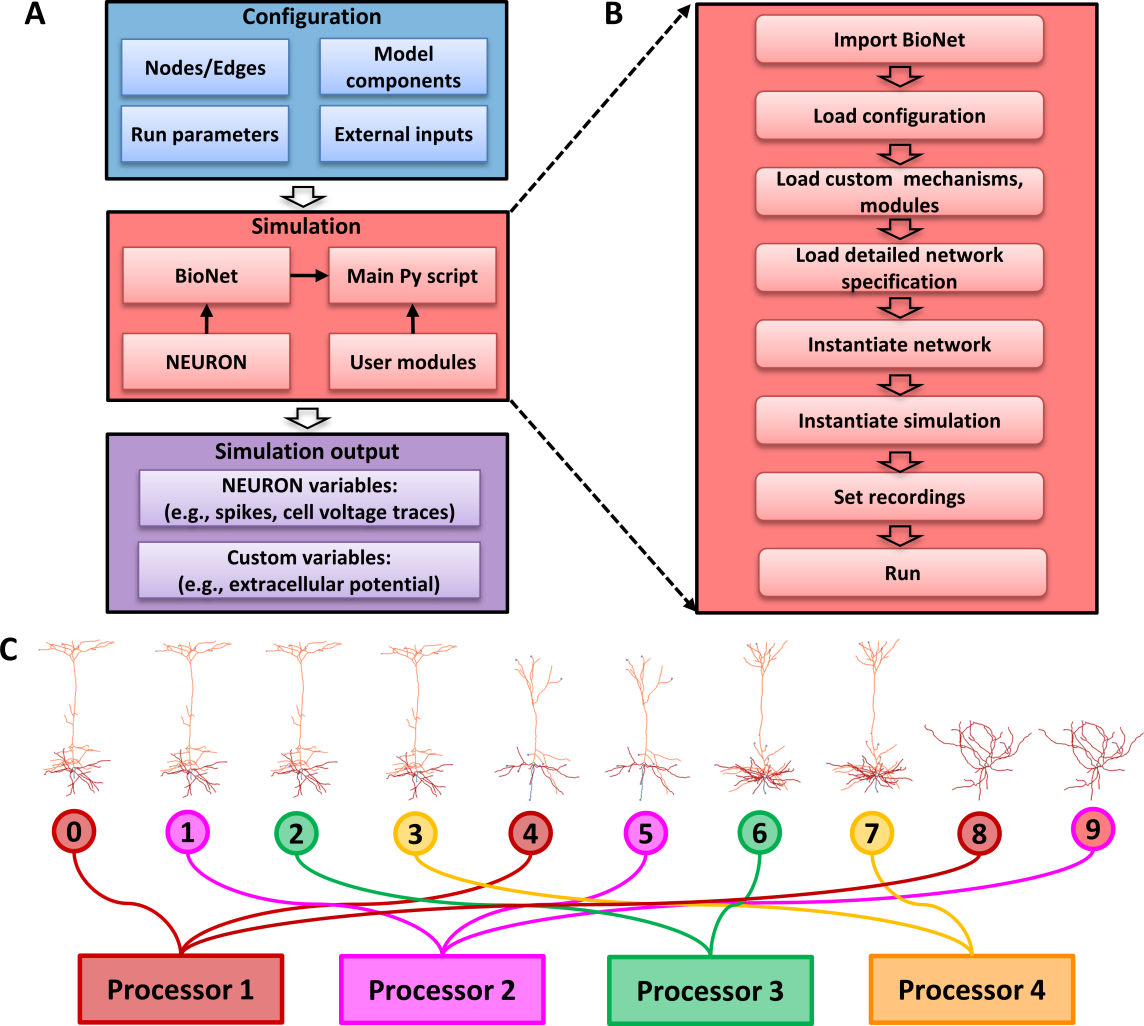
Running simulations. (**A**) Relationships among various elements involved in running simulations with BioNet. The pre-built network (blue), is passed to the main Python script (pink) that loads custom user modules and runs BioNet/NEURON to produce the simulation output (purple). (**B**) The stages of the simulation executed by the main Python script. (**C**) Algorithm for distributing the cells over a parallel architecture. This simple example shows 10 cells distributed across 4 parallel processes (typically each parallel process corresponds to a CPU core). Cells are assigned to each process in turn (a “round-robin” assignment).

To run a simulation, the user first needs to prepare a configuration file specifying several run parameters (time step, simulation duration, etc.) as well as the paths to the files describing the network model, its components, recording electrodes and the desired output directories (Fig 4A). The configuration file (specified in JSON format,) is provided as a command line argument when executing the main Python script:

> python run_my_sim.py my_config.json

The main script calls the API’s functions and custom user modules loading the network description, instantiating cells, connecting them, setting up desired recordings, and running the simulation (Fig 4B). More details for running simulations are provided in the online tutorial.

BioNet supports running simulations on parallel architecture via NEURON’s Parallel Context library [15]. Briefly, the cells are distributed across M host processors in a circular (Round-robin scheduling) manner such that the k-th parallel process will simulate cells with IDs: k, k+M, k+2M, etc., thus hosting ~ 1/M-th of all cells. This pattern allows one to distribute the computational load approximately evenly, especially for networks with a large number of cells, even when they include highly diverse cell models (Fig 4C).

### Simulation output

BioNet provides a flexible and customizable functionality for outputting variables computed by NEURON (e.g, transmembrane voltage) as well as computing custom output variables (e.g., extracellular potential) not provided by NEURON. The properties of the output variables are described in the configuration file that specifies sections, node_ids, the name of the output file as well as the Python classes that implements the output of each variable. BioNet provides a few built-in classes that implement the output of the somatic membrane voltage, intracellular somatic calcium concentration, spikes and the extracellular potential. This functionality is enabled in BioNet by interfacing into NEURON’s standard run system allowing calls to user-defined classes at different stages of the simulation: at the beginning of the simulation, on each time step and when the simulation is completed. These classes serve as templates for users to define additional user-specific simulation outputs.

At each time step, simulation results are stored to a memory block and written into HDF5 files every N steps, as defined by the user in the configuration file. This gives users the ability to monitor the state of the simulation continuously (rather than waiting for the simulation to finish), and to analyze results while the simulation is in progress. Importantly this avoids filling the random-access-memory with recorded variables for long simulation runs.

During simulation, efficient data output is attained by each processor saving data independently into a temporary dedicated file in the centralized storage, and then, at the end of the simulation, combining data across the files generated by each processor. For spikes the data is simply merged into a single larger file. For extracellular potential recordings on a set of electrodes the contributions from all cells are added together in order to arrive at the cumulative extracellular potential.

### Application Example: model of the Layer 4 of mouse V1

**Fig 5:**
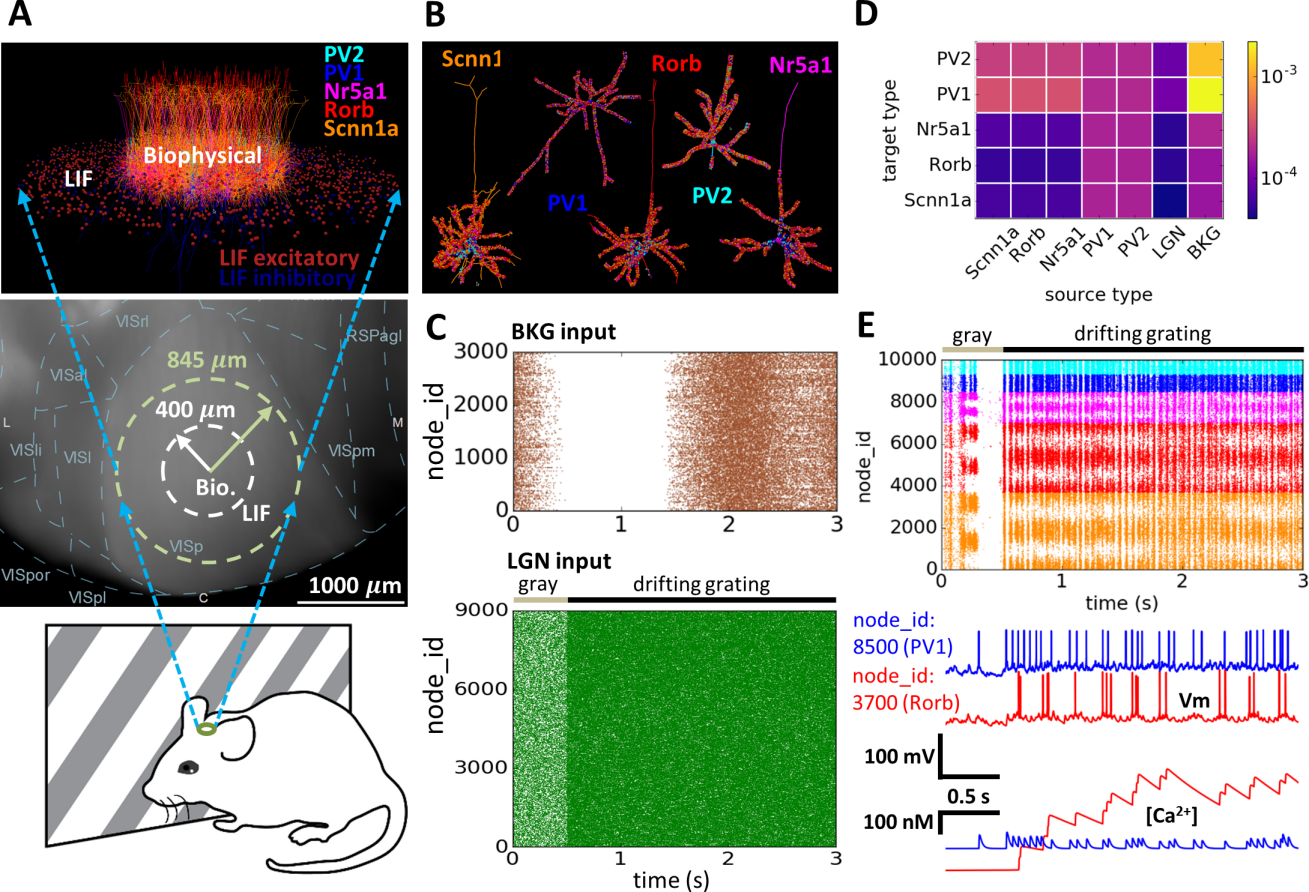
Application example: Model of the layer 4 in mouse V1. (**A**) The *in silico* study [9] mimicked *in vivo* visual physiology experiments (bottom), where a mouse watches visual stimuli such as, e.g., drifting gratings, while the activity of neurons in its cortex are recorded. (Center) The top view of the cortical surface, with boundaries of cortical areas delineated (VISp is V1). The inner boundary encloses part of the tissue that was modeled using biophysically detailed cells, whereas the tissue between the inner and outer circles was modeled using the simplified LIF cells. (Top) The 3D visualization of the layer 4 model (only 10% of cells are shown for clarity). (**B**) Example of synaptic innervation of the biophysically detailed cell models of each type. Synapses (depicted as spheres) are color coded according to their source cell type. (**C**) Rastergrams of the external inputs: (Top) “background” input (BKG, khaki) that switches between “ON” to “OFF” states, loosely representing different brain states; (Bottom) LGN input (green) corresponding to the visual response to 0.5 second gray screen (gray line) followed by 2.5 second drifting grating (black line). (**D**) The connection matrix showing the peak conductance strength for connections between each pair of cell types. (**E**) Simulation output: (Top) spike raster in the biophysical “core”. The node_ids are ordered such that cells with similar ids have similar preferred orientation angle. In this example, cells preferring ~0, ~180, and ~360 degrees are responding strongly to a horizontal drifting grating. (Bottom) somatic voltage traces and the corresponding calcium traces for example excitatory (red) and inhibitory (blue) cells.

We demonstrate the application of BioNet for simulating activity of a network of the input layer 4 in mouse V1 receiving LGN input [9], which was constructed and simulated using an early development version of BioNet. The model is composed of a cylindrical “core” domain (radius 400 *μ*m) within layer 4 (height 100 *μ*m) in the central portion of V1 and includes 10,000 biophysically detailed cells (Fig 5A). The model also includes 35,000 leaky integrate-and-fire (LIF) cells within the “periphery” annulus (outer radius of 845 um), so that the biophysical cells at the border of the “core” have on average the same number of neighboring cells to connect with as those at the center. The biophysically detailed core includes 5 “cell types”: three excitatory, as determined by major layer 4-specific Cre-lines (Scnn1a, Rorb, Nr5a1, 85% of all cells), and two inhibitory, parvalbumin-positive fast-spiking interneurons (PV1 and PV2, 15% of all cells). Each “cell type” is modeled by a single cell model from the Allen Cell Types Database [21], and uses a compartmental model for the reconstructed dendritic morphology with active conductances at the soma and passive conductances at the dendrites (Fig 5B). The LIF cells on the “periphery” are modeled using one excitatory and one inhibitory “cell type” model. The connection weights and innervation domains depend on the pre- and post-synaptic types (Fig 5B and 5D). The connectivity in the network differed by cell type, distance, and orientation selectivity of neurons. In particular, one objective of this work was to test the role of the so-called like-to-like connectivity in the cortex, where cells preferring similar orientations of the grating stimuli are more likely to connect to each other than cells with different preferred orientations [26–28]. Our builder functionality was important for enabling these complex and heterogeneous connectivity rules.

The network receives two kinds of feed-forward external inputs (that were pre-computed separately from the simulation of the V1 network): from the lateral geniculate nucleus (LGN) and a “background” (BKG) (Fig 5C). The LGN input is triggered by visual stimulation (first 0.5 second: gray screen; remaining time: a drifting grating like the example in Fig 5). The BKG input represents the input from the rest of the brain and alternates between “ON” and “OFF” states, accounting for different brain states. Both LGN and BKG activity are modeled with firing rate models that produce firing rates of individual cells that were subsequently converted to spikes utilizing Poisson process. A key advantage conferred by BioNet was that once a few instances of the V1, LGN, and BKG populations were constructed and saved to file, they could be easily mixed and matched to provide a large number of simulation configurations and trials, without the need to re-build connectivity or re-generate spikes from LGN and BKG for each new simulation trial.

The corresponding simulation output for the biophysical “core” of the layer 4 model with the spike responses as well as somatic voltage and [Ca^2+^] traces of several selected cells are shown in Fig 5E. The spiking activity demonstrates the prominent orientation selectivity of excitatory but not inhibitory cells, and oscillations in the ~20 Hz range, consistent with extracellular electrophysiological experiments [9]. Analysis of the simulation output conducted in the dedicated study [9] reveals that the model reproduces several findings from *in vivo* recordings, such as magnitude of responses to gratings, orientation selectivity, prominence of gamma oscillations, long-tailed distributions of firing rates, lifetime sparsity [29] for gratings and movies, the magnitude of cortical amplification, and others. Furthermore, the model makes predictions regarding the effect of optogenetic perturbations of the LGN or the Scnn1a population in layer 4 as well as the effects of the different connectivity rules on the observed activity [9].

### Application Example: Computing extracellular potential

Extracellular potential is a major observable in electrophysiological experiments in vivo. The high-frequency component of the signal, the multi-unit activity (MUA), provides information about the spiking activity of neurons in the vicinity of the recording electrode contacts [30] and the low-frequency component, the local field potential (LFP), contains information about the collective coordinated activity of neuronal populations [31,32]. Recent advances in the multi-electrode fabrication for high density and high throughput recordings [33,34] led to increasing interest in the problem of interpreting extracellular potential recordings in terms of underlying neuronal activity, with modeling as an essential approach [35,36].

NEURON’s built-in extracellular mechanism allows one to account for the properties of the extracellular space. However, this mechanism alone does not support computation of the extracellular potential at electrode sites receiving contributions from many nearby cells. A recently developed Python tool LFPy [37] allows calculating extracellular potential from biophysically detailed single cells and populations of unconnected cells simulated with NEURON. It was successfully applied for modeling extracellular potential recordings with multielectrode arrays *in vitro* [38] and *in vivo* [39,40] from the populations of biophysically detailed cells with the network activity pre-computed by an external simulator (e.g., NEST [41]).

**Fig 6:**
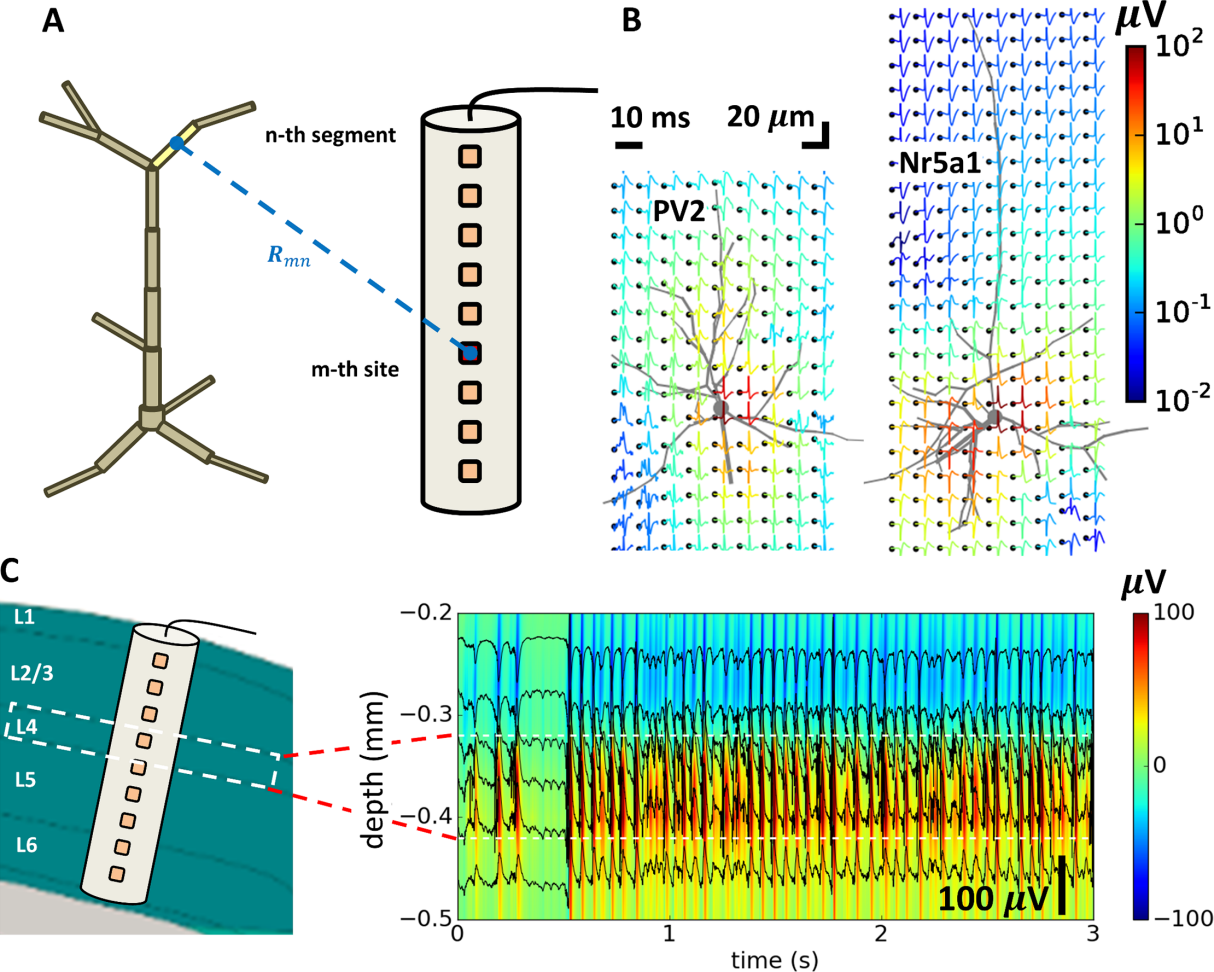
Computing extracellular potential. (**A**) Schematic of the compartmental model of a cell in relationship to the recording electrode. The calculation of the extracellular potential involves computing the transfer resistances *R_mn_* between each n-th dendritic segment and m-th recording site on the electrode. (**B**) Extracellular spike “signatures” of individual cells recorded on the mesh electrode (black dots), using two single-cell models from the layer 4 network model as examples: PV2 (left) and Nr5a1 (right). (**C**) Modeled extracellular recordings with the linear electrode positioned along the axis of the cylinder in the layer 4 model (left). Extracellular potential responses (right) show all simulated data (color map) as well as from six select channels (black traces superimposed on the color map).

BioNet enables further advances in the field by providing the capability for computing extracellular potentials from concurrent network simulations of biophysically detailed neurons in a self-consistent manner. The extracellular potential is computed using the line-source model [42]. It is further assumed that membrane current is uniformly distributed within individual computational compartments (i.e., NEURON segment) and that the extracellular medium is homogeneous and isotropic. The extracellular potential at the m-th location (i.e., electrode site) generated by a single cell is thus computed Φ_*m*_ = ∑*R_mn_I_n_*, where *I_n_* is the trans-membrane current passing through the n-th segment and *R_mn_* is the transfer resistance between n-th segment and the m-th electrode site (Fig 6A). The transfer resistances are computed using expressions that assume a uniform current distribution within individual segments [43,44]. The contributions of individual cells to the recordings (Fig 6B) are then summed up to find the total recorded potential from all cells at each recording electrode site. The example layer 4 model (see Sec. “Application Example: Layer 4 model of the mouse V1”) presented above was also recorded with a virtual linear electrode positioned at the center of the network and perpendicular to the cortical surface and is shown in Fig 6C. Extracellular potential traces show the local field potential (LFP) manifested as low-frequency oscillations present across all electrode channels resulting from the collective neuronal activity in the network. Riding on top of the low-frequency LFP, traces also show the extracellular action potentials—manifested as sharp downward deflections—representing spikes from individual cells, whose somata are positioned sufficiently close to the recording sites. This capability of BioNet was utilized to simulate *in silico* ground truth extracellular recordings in order to test different electrode designs [34] and validate and improve spike-sorting algorithms [45].

### Computational performance

**Fig 7:**
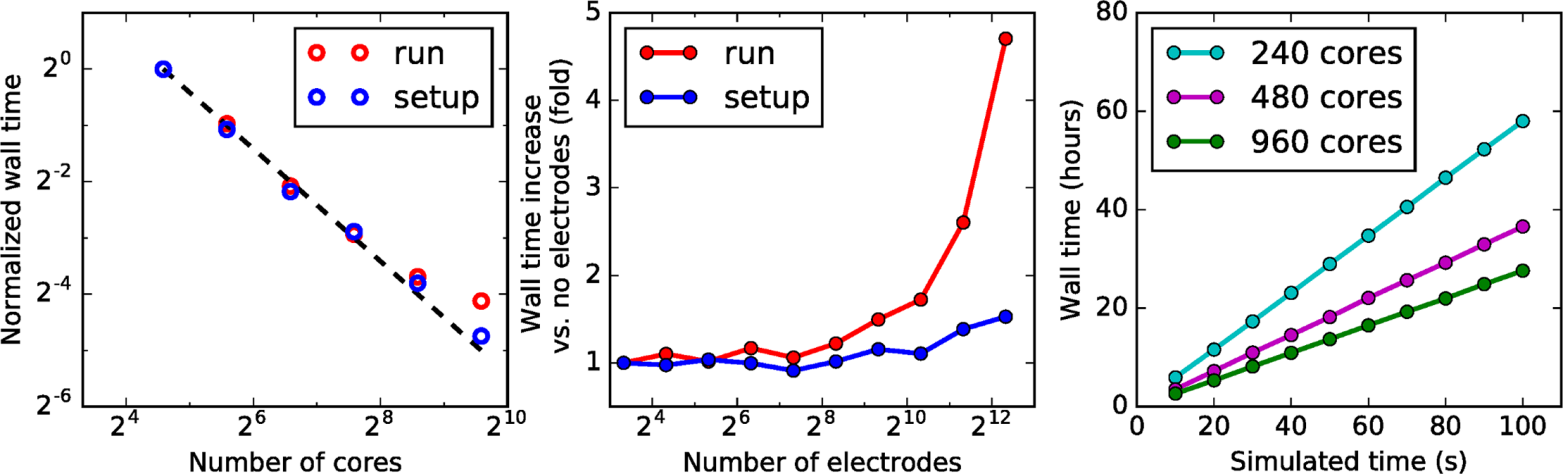
Computational performance. (**A**) Scaling of wall time duration (normalized by the duration on a single CPU core) with the number of CPU cores for the simulation set up (blue circles) and run (red circles) of the layer 4 model (see Fig 5). The ideal scaling is indicated by the dashed line. (**B**) Wall time increase when computing the extracellular potential for both set up (blue circles) and run (red circles) durations. (**C**) Scaling of the wall time with the simulated time for a long simulation. The non-ideal scaling with the increase in the number of cores corresponds to the deviations from the dashed line in (A).

We evaluated the run time performance of the simulations using the layer 4 model (see Sec. “Application Example: Layer 4 model of the mouse V1”). The simulations were performed on various numbers of nodes each including 24 hyper-threading cores (12 actual cores) of Dual Intel Xeon CPUs E5-2620 with clock speed of 2.00 GHz and 132GB RAM. Each node includes 2x 1000GB Hard Disks with CPUs connected via 1 Gb Ethernet. The wall time (that would be recorded by a wall-clock) for both set up and run scales nearly ideally for up to ~1000 CPU cores (Fig 7A). The slight loss of performance for large number of cores is in part attributed to NEURON’s increased work load involved in the internal communication between processors [14].

The computation of extracellular potential adds only a small increase to the wall time for electrodes with up to ~1000 recording sites (Fig 7B). This is achieved by utilizing NEURON’s cvode.use_fast_imem() function, which returns the transmembrane currents on each time step without needing to utilize NEURON’s computationally expensive EXTRACELLULAR mechanism. However, for larger numbers of recording sites the matrix multiplication involved in computing the extracellular potential begins to dominate the computational burden. Thus, for number of sites greater than ~1000, this results in nearly doubling of the wall time when doubling the number of recordings sites.

The wall time scales linearly with the duration of the simulation, which allows longer simulations to be run (order of minutes of simulated time). As we show in Fig 7C, test simulations of the layer 4 model were carried out for 100 seconds of simulated time, while the compute time in these tests was strictly linear as a function of the simulation time.

## Discussion

The Python-based API to NEURON developed here is designed to free neuroscientists’ efforts from the need to write idiosyncratic, narrow-purpose modeling code and instead empower them to carry out systematic, large-scale high-throughput simulations to address scientific questions. BioNet enables functionality for building custom network models, described in a set of files and subsequently loading them to be seamlessly simulated with NEURON on parallel hardware. BioNet provides a Python interface facilitating the application of both NEURON’s built-in and custom (e.g., previously published) models of cells and synapses. It provides users with the capability to execute custom run-time calculations similarly to how extracellular potential calculations were implemented (see Section “Computing extracellular potential”). BioNet is publicly available (github.com/AllenInstitute/bmtk) with accompanying documentation and tutorials hosted at alleninstitute.github.io/bmtk/.The challenges involved in constructing and simulating models with NEURON led to the introduction of a number of software tools designed to facilitating modeling neuronal networks. These include tool suites such as NeuroConstruct [17], PyNN [18] and NetPyNE (neurosimlab.org/netpyne/), each of which has been designed with a particular set of applications in mind. NeuroConstruct is a graphical user interface designed to simplify development of the biophysically-realistic small-scale models (that can be simulated on a single processor). It converts user specifications into simulation scripts that can be executed on a variety of simulator backends. In contrast, PyNN is a Python application programming interface designed to enable modeling at a high level of abstraction that supports parallel simulations for several simulator backends. While originally developed for simplified neuronal models, it is now expanding to also support the morphologically-detailed models. NetPyNE is designed as a dedicated suite of tools facilitating development, analysis, and parallel simulation of large biophysically detailed networks with NEURON. It utilizes hierarchical data structures for both the high-level specification of networks as well as for instantiated networks that can be stored to files for subsequent simulations.

In turn, BioNet was specifically designed to address the challenges faced when developing large-scale biophysically detailed networks that are very computationally expensive to construct and simulate. BioNet addresses these challenges by utilizing a modular approach to modeling, where building and simulation stages are distinctly separated. Thereby a network can be built in incremental steps and saved in files for subsequent refinement and simulation. This capability is supported in BioNet by describing properties of nodes and edges using a relational data model [46] that enables a highly independent and parsimonious representation of network parameters. Thus, small changes to a model require small effort on user’s part, enabling rapid hypothesis testing regarding the role of various structural components on network function.

The modular interface design and capabilities of BioNet should help address the current challenges of model sharing and reuse present in the computational neuroscience community. We believe that the adoption of such a modular workflow—whereby the building, simulation and analysis tools are fully independent and interface via file exchange—will encourage wider application of biophysically detailed modelling. This approach has been very successful, for example, in the field of molecular dynamics simulations of proteins, where tools for model building, simulation, and visualization interface with each other via commonly supported file formats [47–51]. In turn, this will lead to the advancement and adoption of the common network descriptions interfacing these tools, which has been lagging behind (although some promising initiatives are underway, e.g., NeuroML [24]). Taken together, these efforts will empower neuroscientists to develop reusable large-scale, data-driven biophysically detailed network models and make large-scale modeling accessible and appealing to a much broader range of neuroscientists.
